# An overview of microalgae biomass as a sustainable aquaculture feed ingredient: food security and circular economy

**DOI:** 10.1080/21655979.2022.2061148

**Published:** 2022-04-07

**Authors:** Ashfaq Ahmad, Shadi W. Hassan, Fawzi Banat

**Affiliations:** Department of Chemical Engineering, Khalifa University of Science and Technology, Abu Dhabi, United Arab Emirates

**Keywords:** Aquaculture, microalgae, aquafeed, biochemical composition, sustainability, bio-economy

## Abstract

Sustainable management of natural resources is critical to food security. The shrimp feed and fishery sector is expanding rapidly, necessitating the development of alternative sustainable components. Several factors necessitate the exploration of a new source of environmentally friendly and nutrient-rich fish feed ingredients. Microalgal biomass has the potential to support the growth of fish and shrimp aquaculture for global food security in the bio-economy. Algal biorefineries must valorize the whole crop to develop a viable microalgae-based economy. Microalgae have the potential to replace fish meal and fish oil in aquaculture and ensure sustainability standards. Microalgae biomasses provide essential amino acids, valuable triglycerides such as lipids, vitamins, and pigments, making them suitable as nutritional supplements in livestock feed formulations. Fish and microalgae have similar nutritional profiles, and digestibility is a critical aspect of the aquafeed formulation. A highly digestible feed reduces production costs, feed waste, and the risk of eutrophication. Due to low input costs, low carbon footprint, wastewater treatment benefits, and carbon credits from industrial CO_2_ conversion, microalgae-based fish and shrimp feeds have the potential to provide significant economic benefits. However, several challenges must be addressed before microalgal biomass and bioproducts may be used as fish feeds, including heavy metal bioaccumulation, poor algal biomass digestion, and antinutrient effects. Knowledge of biochemical composition is limited and diverse, and information on nutritional value is scattered or contradictory. This review article presents alternative approaches that could be used in aquaculture to make microalgal biomass a viable alternative to fish meal.

## graphical abstract

**Figure:**
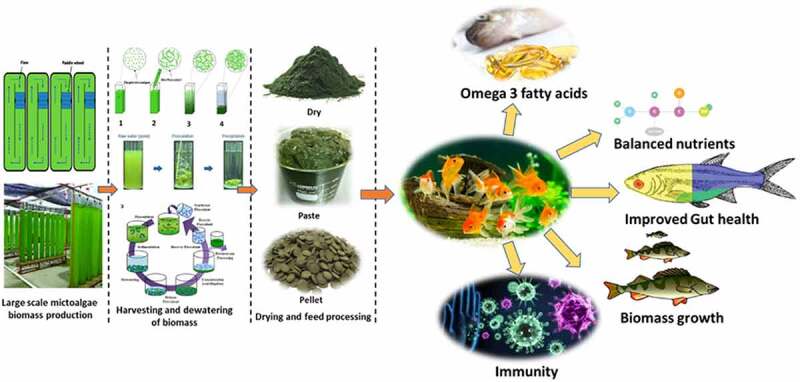

## 1. Introduction

Aquaculture has become increasingly important for food security in the 21^st^ century. By 2050, the global population is projected to increase from 7.6 to 9.8 billion, resulting in a 60 to 100% increase in food consumption [1,2]. Fish meals, which are high in protein, are an excellent source of nutrients for fish and shrimp in aquaculture systems [3,4]. However, volatility in the global market for fish meals has harmed long-term revenue and security in the aquaculture sector [3,5]. In recent years, the gradual exhaustion of marine fisheries resources has posed a serious problem for fish meal production [6]. In China, for example, marine capture increased more than 20-fold from 0.6 million tons in 1950 to 13.6 million tons in 2011. Additionally, periodic closures and efforts to manage fishing capacity in the offshore ocean have significantly reduced fisheries productivity, thereby limiting fish meal production [7]. Finally, industrialization and urbanization-related marine pollution contaminate fish products and affect the safety of fish meals [8]. Aquaculture is one of the fastest-growing sectors of the food industry. In 2019, the aquaculture industry was expected to be valued at US$ 31.94 billion [9]. The aquaculture industry is expected to grow at a rate of more than 7.1% between 2020 and 2027. Increased human consumption and commercial acceptability are now driving the growth of the aquaculture industry.

In recent years, several new species have been introduced into the industry. Aquaculture has grown faster than other major food industries in recent years due to increased fish consumption. It has been demonstrated that a combination of two microalgal species, *Nannochloropsis oculata*, and *Schizochytrium* sp., can be used to feed Nile tilapia (*Oreochromis niloticus*), the second-largest farmed fish in the world. This study demonstrated that microalgae-based feeds can enhance nutritional quality and fish growth metrics [10]. In another study, *Nannochloropsis* sp. and *Isochrysis* sp. were used to substitute fish meals and fish oil in the diet of rainbow trout (*Oncorhynchus mykiss*), a key model species for salmonid aquaculture. Compared to fish meal and fish oil, Isochrysis sp. was found to significantly increase apparent digestibility coefficients for crude proteins, amino acids, lipids, and fatty acids [11]. Several commercially available algae products, including Verdemin (*Ulva ohnoi*) and Rosamin (*Entomoneis* spp.), were evaluated as potential feed components for Atlantic Salmon (*Salmo salar*). Verdemin and Rosamin were found to have no significant effects on the growth or feeding efficiency of Atlantic Salmon at doses of 2.5 and 5.0%, respectively. However, a significant increase in long-chain omega-3 polyunsaturated fatty acids (n-3 LC-PUFA) was observed in fish fed 5% Rosamin [12]. Therefore, a plant-based fish meal has become an increasingly popular option in recent years.

Improved fish nutrition can reduce feed waste, resulting in enhanced financial sustainability. A diet rich in omega-3 fatty acids, antioxidants, and prebiotics has been shown to increase the production, duration, and quality of farmed fish [13]. Furthermore, supplementing animal feed with algae improves growth and weight gain, decreases feed consumption, increases immunological response, resistance to illness, antibacterial and antiviral activity, and enriches livestock products with bioactive components [14]. Microalgae-related biotechnologies and bioproducts have been rapidly developed in recent years. Research in microalgae has previously focused on improving biomass harvesting efficiency and producing specific high-value compounds in algal cells [15,16]. The rapid growth of the algal bio-economy has been driven by significant advances in algal biotechnology that have turned algae into an efficient ‘cell factory’ for food production [14]. The cost of microalgal feed remains higher than that of conventional feed. The cost of microalgal feed must come down to be competitive. Algal biotechnology is closely related to the growth of the algal bio-economy in terms of food and feed production. Algal biotechnology focuses on increasing algal productivity to reduce the cost of biomass production. Several recent biotechnological approaches have resulted in increased biomass production and accumulation of useful metabolites . These include bioreactor design, production of genetically modified strains, high-throughput screening, rapid sampling, and genetic and metabolic engineering. Microalgal biotechnology focuses on improving the production of carbohydrates, proteins, polyunsaturated fatty acids, pigments, and other valuable nutrients from microalgae through strain optimization, carbon flux alterations, stress condition modifications, and metabolic pathway prediction. In recent years, biotech and bioengineering techniques have enabled algae to become more efficient ‘cell factories’ for carbon sequestration and food production [14,17].

**Figure 1.**
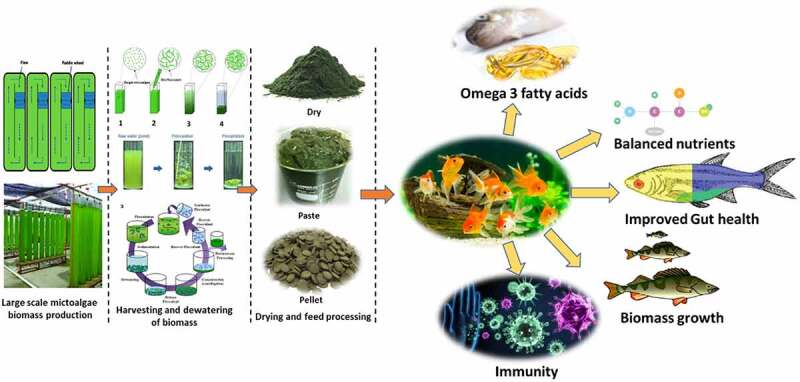
Technology process lineup for the production of beneficial fish-derived food by using an algae-based feed.

Microalgae biomass has been proposed as a high-value feed for fish and shrimp in sustainable aquaculture [18,19]. Table 1 outlines the benefits and drawbacks of a variety of fish-based alternatives to traditional meals. The idea of employing microalgae biomass and bio-products as aquafeed for fish and shrimp growth is novel; nevertheless, various obstacles must be overcome before the concept can be successfully applied. Several issues need to be resolved, including potential safety issues, antinutritional factors (ANFs), limited digestibility, and others. There is a significant amount of cellulose in microalgal cells, which can affect the digestion of algal biomass in fish diets [20]. Microalgae cells, which contain a range of negative charges, have been found to have a substantial potential for the adsorption and accumulation of heavy metals (HMs) [21]. As a consequence of the food chain, these harmful components will concentrate on aquatic animals, endangering the health of humans who consume fish and shrimp. The majority of previous research focused on the benefits and downsides of microalgae biomass in aquafeed. Aquafeed accounts for at least 75%-90% of aquaculture<apos;>s operational expenses. New feed additives are needed, as traditional feed materials such as fish meal, fish oil, and soybean meal have become unsustainable. Aquafeed made from microalgae is not only ecologically beneficial, but with appropriate optimization, it may also be commercially feasible. Microalgae also have a nutritional profile that is comparable to that of many fish. The digestibility of the feed is an important issue to consider when formulating it. A highly digestible feed can help reduce production costs, waste, and the risk of eutrophication in the environment. This article discusses the digestibility of numerous microalgae in fish, as well as approaches to enhance microalgal digestibility. Figure 1 represents the technology process lineup for the production of beneficial meals derived from fish using the algae-based feed.

**Table 1.** Presents the benefits and drawbacks of an alternative fish diet

Alternate Feed	Benefits	Drawbacks	Ref.
Guar meal	Guar meal may be substituted for soy meal without harming growth in certain fish.	This product includes antinutritional and anti-digestive substances such as residual gum, saponin, phytate, and protease inhibitor tannin. Gastrointestinal evacuation is slow. Indigestible amino acids. Oil production and guar gum usage affect the availability of guar meals.	[22,23]
Macroalgae	Bioactive compounds from macroalgae can help farmed fish.	Complex polysaccharides are indigestible Excess heavy metals The probable presence of phlorotannins, lectins, phyto acids, trypsins, and amylase inhibiting substances	[24]
Yeast	Lignocellulosic waste can grow Yeast protein is beneficial for fish, except for its low methionine content. Due to a rapid increase	Yeast protein is low in sulfur-containing amino acids such as methionine and cysteine.	[25,26]
Insects	Food waste may be used as a source of nutrients	Most insect diets were lacking in methionine and cysteine. Antinutritional chitin is reported.	[27]
Blood meal (cow blood)	The protein content is high. Lysine-rich	Methionine deficiency Protein digestibility is greatly influenced by heat sensitivity and drying conditions.	[28,29]
Hydrolyzed feather meal	Hydrolyzed feather meal is rich in cystine (74–61%) and protein.	Not easily digested Low in lysine and methionine (2 % of the crude protein) (1 % crude protein)	[30,31]
Wheat	Protein content is low (11%).	Wheat<apos;>s high starch content makes it a primary source of energy (usually more than 70%). There is a deficiency of lysine.	[32,33]
Microalgae and Algal oil	Microalgae<apos;>s rapid rate of growth A broad selection of species is available, each with a unique set of characteristics. ω-3 fatty acid-rich Contains antioxidants, colorants, and has a probiotic impact	Formulated feeds have a high production cost. Microalgae with rigid cell walls are difficult to digest.	[34,35,40]

## 2. Microalgae as fish meal

**Figure 2.**
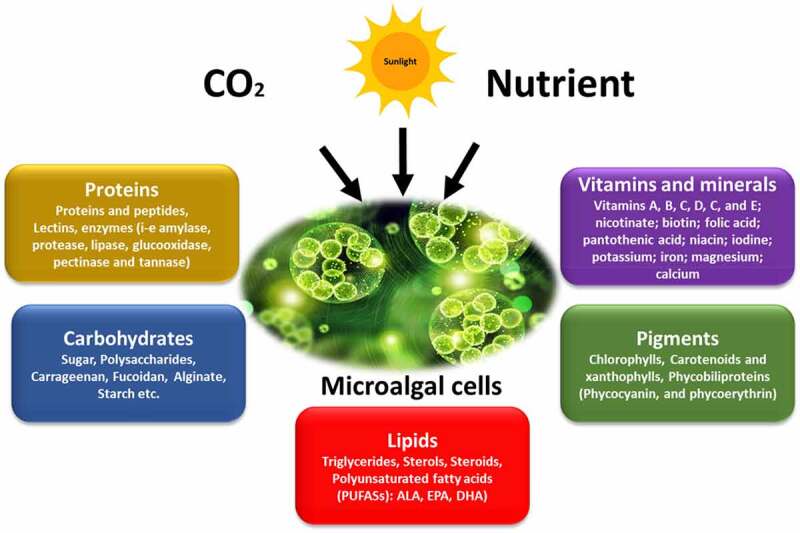
Metabolites produced by microalgae during their photosynthetic activity.

Microalgae are photosynthetic microorganisms that utilize atmospheric carbon dioxide (CO_2_) and sunlight energy to produce a variety of proteins, carbohydrates, lipids, minerals, vitamins, polyphenols, flavonoids, and carotenoids, as shown in Figure 2. Microalgae-based products can be used in a variety of industries, including food and beverages, animal feed, cosmetics, chemicals, and biofuels. By 2028, the market for microalgae-based products is expected to grow from US$ 1,547.23 million in 2020 to US$ 2,811.10 million. It is expected to grow at a compound annual growth rate (CAGR) of 7.9% between 2021 and 2028 [36]. To date, the microalgae sector has focused mainly on species that are associated with food and cosmetics. Microalgae species, including *Spirulina* sp., *Dunaliella* sp., *Isochrysis* sp., *Pavlova* sp., and others, are also used as larvae feed by fish hatcheries, although these are not normally grown on a large scale. However, in recent decades, microalgae have been explored as a possible bulk-feeding ingredient for fingerlings and adult fish [37]. Microalgae have been suggested as a substitute for fish food for several reasons. Microalgae have the highest net biomass productivity compared to any terrestrial plant or animal [38].

Microalgae, unlike land-based plants, do not need fertile soil to grow. Microalgae can even grow in seawater or wastewater [39]. Current land-use patterns do not require large-scale microalgae cultivation on non-arable land or non-potable water (or practices). Unlike insects and bacteria, microalgae have minimal nutritional requirements. In a biorefinery, microalgae might be utilized to produce fish feed [40–42]. The promise of microalgae is based on its protein, lipid, carbohydrate, and pigment composition, which is ideal for fish health. Some economically relevant microalgal species have chemical compositions that are equivalent to feed components utilized in the aquafeed industry (Table 2). Methionine, for example, is abundant in microalgae, unlike plant-based components such as *Chlorella* and *Chlamydomonas* [43]. The proportion of starch in microalgal species varies from 7 to 45% [44]. Other microalgae contain less starch (30–49%) than *Tetraselmis subcordiformis, C. rheinhardtii*, and *C. vulgaris* [44].

**Table 2.** Nutrient content of several microalgae species

Microalgal strains	Composition (%)			Ref.
	Lipids	Protein	Carbohydrates	
*Haematococcus pluvialis*	15	48	27	[103]
*Dunaliella*	25–75	50–80	10–25	[61,62]
*Botryococcus braunii*	33	39.61	2.38	[63]
*Nannochloropsis sp.*	22–31	33–44	8–14	[155]
*Botryococcus*	25–75	3–10	17–21	[64,65]
*Scenedesmus quadricauda*	1.9	47	21–52	[156]
*Chlamydomonas*	20–25	47–50	15–20	[66,67]
*Synechococcus sp.*	11	63	15	[68]
*C. vulgaris*	14–22	12–17	1–58	[69]
*Arthrospira platensis* (*Spirulina*)	7–23	57–65	20–30	[70,71]
*Isochrysis galbana*	12–14	50–56	10–17	[155]
*Porphyridium cruentum*	5.78–7.55	27.7–40.8	22.8–39.3	[72]
*Spirulina maxima*	6–7	60–71	13–16	[155]
*Tetraselmis maculata*	3	52	15	[156]
*Nannochloropsis granulata (CCMP-535)*	33.5	23.6	36.2	[73–75,182]
*Phaeodactylum tricornutum (CCMP-1327)*	18.2	39.6	25.2	
*Arthrospira platensis*	14.2	55.8	22.2	
*Tetraselmis chuii (PLY-429)*	12.3	46.5	25.0	

Next-generation microalgae-based feeds have the potential to provide a sustainable source of aquaculture food. In addition to providing essential nutrients, microalgae are an essential food source for zooplankton and lower trophic fish, which in turn provide food for fish at higher trophic levels. Microalgae can contain up to 60% protein, 60% carbohydrates, or 70% oil, depending on the species of algae and its growing conditions. Secondary metabolites generated by microalgae, such as pigments, growth-promoting compounds, and hormones, have intrinsic antioxidants, antibacterial, anti-inflammatory, and immune-stimulant properties that benefit both marine and freshwater species [19,45–47]. Furthermore, numerous species can produce eicosapentaenoic acid (EPA), docosahexaenoic acid (DHA), and colors (e.g., carotenoids) from the ground up, proving their adaptability. In the future, the cultivation of microalgae on non-arable land or along coastlines may greatly increase the global photosynthetic primary production by lowering water demand and recycling nutrients by using wastewater and seawater, as well as converting atmospheric CO_2_ into nutrient-dense feed and animal health products. In this sense, a circular aquaculture firm could arise as part of the larger circular bio-economy [19,48–50]. In addition, the contents of EPA and DHA in microalgal lipids are significantly higher and also less contaminated than fish oil. PUFA-rich microalgae include species of *Schizochytrium, Crypthecodinium, Nannochloropsis*, Isochrysis, *Nitzschia, Diacronema, Porphyridium*, and *Desmodesmus*, which produce EPA and DHA, respectively [51,52]. For example, *Nannochloropsis* sp. and *Phaeodactylum tricornutum* have about 39% EPA and 30% DHA of total omega-3 fatty acids, while *Schizochytrium* sp. and *Thraustochytrium* have about 40% DHA and 22% EPA, respectively [53]. Furthermore, microalgae produce antioxidant pigments, and some microalgae contain vitamins and immunostimulants that are beneficial to aquatic organisms [13,54].

Microalgae have the potential to control food production and pollution by assimilating nutrients from water and wastewater [55,56]. Wet effluents from markets and slaughterhouses contain large amounts of nitrogen and phosphorus [57]. Washing fruits and vegetables at public markets or slaughterhouses and washing poultry and fish produces wet market wastewater [58], which has higher levels of nitrogen, phosphate, chemical oxygen demand, biological oxygen demand, fats, solid particles, oils, and greases than domestic wastewater [59]. Algae biomass derived from nutrient recycling of wet market and slaughterhouse effluents can be used as a fish meal. A biorefinery strategy may result in resource-efficient, environmentally friendly value chains with a low carbon footprint through the co-production of bio-based and biodegradable products when algal generation systems are integrated into aquaculture operations (a biorefinery approach). This strategy may also benefit the aquaculture industry and the general public in other ways, such as healthier diets and ecosystem services [48,60].

### 2.1 Microalgae fatty acids

Lipids play a dynamic role in the formation of membranes and are important energy storage molecules. Although microalgae may have oil contents exceeding 60% by weight of dry biomass, the most common oil contents are in the range of 20–50% [76–78]. Polyunsaturated fatty acids present in microalgal lipids include arachidonic acid (ARA) and DHA. *Cryptothecodinium* and *Schizochytrium* contain DHA, *Phaeodactylum, Nitzschia, Isochrysis*, and *Diacronema* contain EPA, and *Cryptothecodinium* and *Schizochytrium* contain ARA. Microalgal species can produce EPA concentrations ranging from 7 to 34% fatty acids. These fatty acids are rare and difficult to synthesize in a lab. These components are currently derived from fish oil and are restricted in vegetable oils, including palm, soybean, rapeseed, and canola, in aquafeed [79]. Microalgae may produce high amounts of lipids and have a nutritionally advantageous fatty acid composition when grown under stress conditions. Polyunsaturated fatty acids (PUFAs), such as EPA (20:5, −3), and DHA (22:6, −3) are the important constituents of microalgal lipids. Microalgae species, such as *S. limacinum, P. tricornutum*, and *Nannochloropsis* sp., comprise 30–40% of ω-3 fatty acids in their total content [53]. LC-PUFAs such as ω-3 and 6 must be consumed regularly because humans and many animals cannot synthesize them [80]. PUFAs are also necessary ingredients for fish growth because they cannot be formed from saturated and monounsaturated fatty acids [81]. Fish have high tropical levels in aquatic environments due to their ability to metabolize PUFA to produce LC-PUFA, which strongly contributes to tropical upgrading. It is critical to understand how farmed fish create and store LC-PUFAs to substitute terrestrial plant lipids for fish oil in commercial aquafeeds. Through enhanced synthesis and selective use of dietary fatty acids, it is possible to increase the body<apos;>s storage of LC-PUFA. Data synthesis was performed to determine optimal fatty acid levels that improve the generation and storage of omega-3 polyunsaturated fatty acids in edible portions of salmonids [82].

Microalgae improved the fatty acid profile of fish and shrimp by improving the ω-3/ ω-6 ratio, increasing PUFA content, and enriching long-chain PUFAs [83,84]. Aquatic animals have higher nutritional value when their fatty acid profile is improved, which benefits consumers. Additionally, supplementing aquatic animals’ diets with microalgal PUFAs can increase their growth and immunity It has been reported that Nile tilapia can digest *Schizochytrium sp*. lipids with 98% efficiency [85]. Lipid digestibility in juvenile European seabass (*Dicentrarchus labrax*), Nile tilapia with *C. vulgaris, Schizochytrium* sp. *Spirulina* sp., and *Chlorella* sp., and African catfish (*Clarus gariepinus*) with *C. vulgaris* and *S. maxima* has been shown to be more than 80% [85–87]. Lower digestibility has been noted under certain conditions, such as for *N. gaditana* in juvenile African catfish [88]. A microalgae-based feed might also improve fish survival due to its functional properties, including probiotics, prebiotics, immunostimulants, antivirals, antibacterials, etc. Bacteria or microbes that are probiotics are believed to contribute to a healthy gut when consumed. For example, microalgae are probiotics for fish. The microbiome in the colon digests algal cells and generates probiotics that inhibit the growth of infectious agents [89,90]. Consumption of *Tetraselmis suecica* live cells significantly reduced the number of harmful bacteria in the stomach of white shrimp (*Fenneropenaeus indicus*) as compared to a control group [91]. The addition of *Schizochytrium* sp. meal to the diet at 1.2% significantly improved Nile tilapia health [92]. Previous studies have found that *Spirulina* can induce non-specific immune responses against infections in a variety of fish [93,94]. When *S. Platensis* is used at a 10% concentration, it has been proven to dramatically increase the production of white blood cells, red blood cells, hemoglobin, albumin, and total protein in rainbow trout [95].

LC-PUFAs are beneficial to animals and humans as they provide biologically active compounds such as prostaglandins and thromboxanes, which are crucial for the formation of cholesterol and triglycerides in the blood, as well as the prevention of certain diseases such as arthritis and rheumatoid arthritis [96]. DHA (22:6) and EPA (20:5), two of the most beneficial LC-PUFAs, stand out for their many health benefits. DHA improves brain health, which maintains neurons, boosts short- and long-term memory, and aids in the treatment of brain disorders such as memory loss and cognitive decline. By reducing oxidative stress and plasma triglycerides, as well as providing benefits for the treatment of inflammation, arrhythmia, and cardiovascular disease [97,98]. Microalgae also produce a wide range of fatty acids that are useful for the food and feed industry, including gamma-linolenic acid (GLA), linoleic acid (LA), alpha-linolenic acid (ALA), and ARA [99]. Wound healing and regeneration, as well as the eradication of invading microorganisms, may be aided by these essential fatty acids [100]. The lipid content of *C. vulgaris* has been reported to be 35–40% by weight, with a content of 27% linolenic and 24% linoleic acid [101]. *Spirulina* sp. has been promoted as an inexpensive source of GLA [100]. Recent research has indicated that microalgae such as *Chlorella* sp. and *Schizochytrium* sp. are more attractive than other autotrophic species due to their nutritional qualities and ability to be effectively consumed by aquaculture species. Thanks to the well-developed technology for mass cultivation of *Schizochytrium* and *Chlorella* sp. in aquafeeds, the use of fish oil can be significantly reduced in the future. To achieve sustainable substitution of fish oil in aquaculture, research must focus on the use of microalgal species.

### 2.2 Proteins and amino acids derived from microalgae

Proteins have been recognized as the building components that are responsible for individual growth. Proteins are constructed from peptide bonds that link amino acid units [102]. In terms of quality and amino acid composition, microalgae protein is a great alternative to fish meals. The protein concentration of algae has been reported to range from 40 to 60 wt/wt % [103]. Another study discovered that *C. vulgaris* has between 51 and 58% protein, while *Spirulina* sp. comprises between 60% and 71%. Additionally, *Arthrosphira platensis* has a protein content of 70% by weight [104]. Protein is so abundant in some microalgae species that it accounts for more than half of their biomass. Most *Spirulina* strains, as well as a few *Chlorella* and *Nannochloropsis* strains, have a protein content of 40 to 65% [105]. Microalgae can synthesize all amino acid molecules; therefore, algae-derived amino acids are preferred over other protein-rich foods [106]. Microalgae can synthesize several protein compounds faster than traditional protein sources. Table 3 summarizes the current research on microalgal biomass used as a replacement for fishmeal and fish oil.

**Table 3.** Microalgal biomass as an alternative or supplement to fishmeal and fish oil

Microalgae species	Aquaculture species	Fish oil/fish meal/dietary inclusion level replacement	Effects of algae biomass	Ref.
*Schizochytrium*	Pacific white shrimp (*Litopenaeus vannamei*)	4% inclusion in the diet	Although shrimp survival, digestive enzyme activity, and fatty acid content were not affected, their specific growth rate was much higher than in the control group.	[118]
*Dunaliella salina*	Giant tiger prawn (*Penaeus monodon*)	5–10% incorporation in feed	The immune system and antioxidants (superoxide dismutase and catalase) improved significantly and the survival rate was significantly boosted.	[119]
*Phaeodactylum* *tricornutum*	Atlantic salmon (*Salmo salar*)	6% replacement of fish meal	There is no negative impact on growth, protein, lipids, energy, ash, growth performance, etc., in the feed that is used.	[109]
*Nannochloropsis sp. and Isochrysis* sp.	Juvenile Atlantic cod (*Gadus morhua*)	15% fish meal protein replacement	Increased feed intake and fish growth. Survival, feed conversion ratios, and muscle ω-3 and ω-6 fatty acid levels did not differ between the treatment groups.	[120]
*Schizochytrium* sp.	Tilapia (*Oreochromis niloticus*)	100% replacement of fish oil	However, the survival rate did not alter substantially.	[85]
*Nannochloropsis gaditana, T. chuii, and Phaeodactylum tricornutum*	Gilthead seabream (*Sparus aurata*)	0.5 and 1% inclusion in feed	Increased defensive activity	[121]
*Chlorella vulgaris*	Giant freshwater prawn (*Macrobrachium rosenbergii*)	6–8% fish meal substitute	*M. rosenbergii* postlarvae had a faster growth rate, a better immunological response (total haemocyte count and prophenoloxidase activity), and were resistant to Aeromonas hydrophila infection.	[122]
*Arthrospira* sp.	Golden barb (*Puntius gelius*)	20% fishmeal substitute	Fish growth rates have increased significantly.	[123]
*Pavlova viridis*	European sea bass (*Dicentrarchus labrax*)	Fish oil replacement 50–100%	In terms of growth performance and nutrient consumption, there are no detrimental consequences on fish.	[124]
*Nannochloropsis* sp.				
*Nanofrustulum* sp.	Atlantic salmon (*Salmo salar*), common carp (*Cyprinus carpio*)	fish meal replacement 5 or 10%	Algal meal outperformed fish meal in terms of growth and feed intake, indicating that it may be used in place of fish meal.	[125]
*Tetraselmis* sp.	Pacific white shrimp (*Litopenaeus vannamei*)			
*Arthrospira platensis*	Nile tilapia (*Oreochromis niloticus*)	0.5–2% inclusion in feed	Enhanced fish health through tissue protection and antioxidant effects	[126]
*Arthrospira* sp.	Tilapia (*Oreochromis niloticus*)	Replacing fish meal by up to 43%	Unlike corn-gluten meal control, there was no deleterious impact on growth or feed consumption.	[242]

Kim et al. [107] discovered that parrotfish (*Oplegnathus fasciatus*) fed 5% *Arthrospira* had significantly higher weight, protein efficiency, and feed consumption compared to the control group fed fish meal. Fish need meals with 30% to 55% crude protein and amino acids tailored to their individual nutritional needs to achieve maximum growth [108]. Sørensen et al. [109], demonstrated that *Phaeodactylum tricornutum* can replace up to 6% of fish meal in the diet of Atlantic salmon (*Salmo salar*) without affecting digestibility, utilization, or growth performance. Protein digestibility of microalgae varies from 50% to 94% in different fish species. Protein digestibility of rainbow trout, Nile tilapia, European seabass, and African catfish has been reported to exceed 80% [110]. In African catfish and Nile tilapia, bead milling improved *N. gaditana* protein digestibility by 16 and 17 %, respectively [86]. Enzymatically processed microalgae digest protein 6% faster than whole-cell *Nannochloropsis oceanica* [40]. Protein digestion was enhanced in a diet containing *Schizochytrium* sp. when organic minerals were added [87]. *N. oceanica* and *C. vulgaris* provided amino acids with higher digestibility than 90% for European seabass and Atlantic salmon [87,111]. *Tetraselmis* sp. exhibited significantly lower mino acid digestibility than juvenile European seabass. Pretreatment would break down larger proteins into peptides and individual amino acids, which increases amino acid digestibility [110].

Threonine, isoleucine, lysine, leucine, methionine, valine, and histidine are essential amino acids that the body cannot produce itself. Therefore, it is important to consume them through foods that contain EAAs, such as tofu, eggs, and fish [112]. Vegans and vegetarians have few options since the majority of plant-based proteins do not meet the EAA profile. To overcome this problem, an alternative source with a balanced protein profile and low cost is required [113]. Protein digestibility of the microalgal protein (S. *platensis* protein concentration) ranges from 87.5 to 97.8% [114]. However, certain algae (510–710 g/kg) have more protein than eggs or soybeans (132–370 g/kg) and have fairly comparable EAAs [115]. Due to their high content of EAA, microalgae are considered one of the best vegan protein sources. It is well known that microalgae contain EAAs and non-NEAAs, both of which have health benefits [113]. NEAAs include amino acids, proline, arginine, glutamic acid, glycine, aspartic acid, tyrosine, cysteine, serine, and glutamic acid are a few examples. The amino acid profile of *C. vulgaris* and *H. pluvialis*, the proportion of NEAAs is around 51% and 49%, respectively [116]. A healthy immune system is influenced by these chemicals, as well as gene expression, antioxidant responses, and cell signaling [117]. Many studies indicate that the amount of microalgal meal that should be added to aquafeed varies depending on the type of algae used and the aquaculture species being fed. It would be beneficial to study the growth capacity of microalgae and identify the variables that influence their effectiveness.

### 2.3 Microalgae-based pigments

The color of microalgae is one of its most distinguishing properties, which is determined by pigments. Microalgae pigments are critical for their nutritional performance in aquaculture. In addition to chlorophyll, microalgae include carotenoids and phycobiliproteins. The *Nannochloropsis* genus contains pigments such as chlorophyll and astaxanthin. Photosynthesis in algae is facilitated by pigments, which are brightly colored chemical compounds. Carotenoids, chlorophylls, and phycobilin are the three primary types of microalgal photosynthetic pigments [127,128]. Microalgae pigments are eye-catching natural colors that include high-value components with health-promoting qualities that include antioxidants, vitamin precursors, neuroprotective, and immunological boosters [129]. These pigments may address the increased demand for natural colors due to health concerns about the adverse effects of synthetic pigments [127,130]. Aquaculture uses a high concentration of carotenoids, such as β-carotene and astaxanthin, due to their vibrant color and antioxidant effects. These molecules have the potential to improve the quality and value of farmed fish, such as salmon and Asian tiger shrimp (*Penaeus monodon*) [131]. Phytochemicals such as astaxanthin and β-carotene are abundantly generated by the microalgae *Haematococcus pluvialis* and *Dunaliella salina* (3–7% wt/wt) in natural abundance [132,133]. Table 4 shows the pigment compositions of numerous algae species, as well as the health benefits associated with them.

**Table 4.** Algal pigment compositions and their health benefits

Microalgal species	Pigments	Health benefits	Ref.
*Haematococcus*	astaxanthin	Pink colored pigment, Antioxidant, Improved disease resistance, faster growth	[134]
*Spirulina*	β-carotene, astaxanthin	Yellow, orange, and red-colored pigment, antioxidants, improved disease resistance, faster growth	[135]
*Phaeodactylum tricornutum*	fucoxanthin	Golden and yellow coloration, Antioxidant, Anti-inflammatory	[136]
*D. saline*	carotenoids	Photo-protection, camouflage, and signaling enhance immune system	[137].
*Chlorella vulgaris.*	fucoxanthin, zeaxanthin, and lutein	Yellow-colored pigment, antioxidant, anti-inflammatory	[182]
*Scenedesmus sp.*	lutein	Greens and orange-yellow, Antioxidant, reduce inflammation	[75]

Microalgal biomass has been shown to affect fish pigmentation. *H. pluvialis* is the most commonly used microalgae specifically for color enhancement. In the aquaculture industry, whole cells and extracts of *H. pluvialis extracts* are used as feed additives (1.5–1.7%) [134]. Several algae species are used as pigments in fish feed. The *Haematococcus* produces Astaxanthin, which gives salmon its pink hue [134]. Additionally, *Spirulina* contains additional carotenoids that ornamental koi and other fish can convert to astaxanthin and other brightly colored pigments [135]. *Phaeodactylum tricornutum* produces large quantities of fucoxanthin, which has been shown to contribute to the golden yellow coloration of gilthead seabreams [136]. Carotenoids are found in a wide variety of products, including natural feed colors, food supplements, vitamin supplements, and health foods. The high concentration of carotenoids in *D. saline* makes it the most popular species for large-scale production (up to 14% dry weight) [137]. Microalgae strains that are commercially feasible for pigment synthesis must meet a series of criteria, including improved nutritional components, non-toxicity, and the presence of digestible cell walls for nutrient absorption [129]. Phycobiliproteins, β-carotene, and astaxanthin are used mainly as colorants, pharmaceuticals, aquaculture, and nutraceuticals. *Chlorococcum* sp. (Astaxanthin, lutein, β-carotene), *D. salina* (β-carotene, zeaxanthin, chlorophylls a, b), *H. pluvialis* (astaxanthin, canthaxanthin, lutein), *Spirulina* sp. (β-carotene, zeaxanthin, phycocyanin, allophycocyanin), *Porphyridium* sp. (phycoerythrin) [138–143].

Natural feed pigments, feed additives, nutrients, and health food products are commonly made from carotenoids. Carotenoids found in abundance in *D. salina* make it the species most often exploited for large-scale production [144]. Carotenes from *Dunaliella* species were shown to improve the health of *L. vannamei* shrimp given high-carotene diets. Coloration and market acceptability can be achieved by supplementing Red tilapia diets with *A. platensis*, a source of pigmentation [145,146]. According to these studies, low amounts of *Arthrospira* or other microalgae can enhance the color and flavor of numerous fish species, such as tilapia. Therefore, additional studies are required to evaluate the impact of various microalgal pigments on commercial aquaculture.

### 2.4 Microalgae-based vitamins

Microalgae are high in vitamins, and vitamin B has been shown to function as a cofactor for mitochondrial enzymes, reducing oxidative breakdown and improving metabolism [147,148]. Microalgae, including *Spirulina* sp., have more Vitamin B12 (127–244 g/g) than plant or animal-based foods. This vitamin helps prevent megaloblastic anemia, which causes fatigue and weakness [149]. Another study found a significant amount of vitamin E (3.7 mg/g) in the *Euglena gracilis* microalgae. Vitamin E has been reported to reduce the risk of cancer, eye disease, heart disease, and other diseases [150]. A high amount of vitamin C content (3.44 mg/g) has been found in the *Eisenia arborea* brown microalgae, which is equivalent to that of mandarin oranges [151]. This antioxidant vitamin is necessary for immune system function, tissue formation, and repair [152].

### 2.5 Carbohydrates derived from microalgae

Microalgae are rich in carbohydrates, and polysaccharides are readily found in both their cytoplasm and chloroplast [153]. Microalgae carbohydrates are used for several reasons, including energy storage and structural components in cell walls [154]. Due to the high photoconversion efficiency, macroalgae such as *P. cruentum* contain carbohydrates (40–57 wt% dry weight), *Prymnesium parvum* (30–33 wt% dry weight), and *S. quadricauda* (21–52 wt% dry weight) [155,156]. For each species of the algal genus, there is a distinct variation in glucose metabolism and content [156,157]. Algae with high carbohydrate yield and sugar content are suitable for human consumption. The culture system and environmental conditions can impact algal production and glucose content [153]. Microalgae contain about 10–25% carbohydrates and their amount varies with culture age and growth conditions [158]. A variety of starches, cellulose, sugars and other polysaccharides are found in microalgae. Bacteria and fungi naturally produce the polysaccharide β-glucan, which is made of D-glucose. These polysaccharides can be found in large quantities in the *Chlorella* sp., microalgae [78,159]. Table 5 displays the monosaccharide compositions of several microalgae.

**Table 5.** Algal monosaccharide compositions

Microalgal strains	Monosaccharide composition (%)				Ref.
	Arabinose	Glucose	Galactose	Xylose	
*S. platensis*	1.4	24.1	16.4	6.1	[165]
*Arthrospira platensis*	–	38.3	36.4	0.7	[166]
*Chlorella sp.*	34	20	41	–	[167]
*Porphyra ochotensis*	–	5.3	30.4	1.2	[168]
*C. vulgaris* *JSC-*6	1.6	54.9	–	2.3	[169]
*C. marina*	37.6	30.3	10.0	–	[166]

Microalgal carbohydrates are digestible according to the type and quantity of carbohydrates in biomass, as well as the kind of fish that consumes microalgal carbohydrates [160]. The carbohydrate digestibility of microalgal species varies between 22% and 83%, depending on fish species [110]. *S. maxima* and *C. vulgaris* were found to have a higher carbohydrate digestibility (greater than 70%) in Nile tilapia [86]. Microalgae contain starch-like carbohydrates that can be easily digested. In vitro research revealed that *C. sorokiniana, Klamath*, and *N. sphaeroides* showed greater carbohydrate digestibility [161]. Studies conducted on Nile tilapia, African catfish, and in vitro studies have shown that *C. vulgaris* has a higher carbohydrate digestibility value than other algal species [86]. Certain microalgal species, such as *Spirulina* sp., *Chlorella* sp., and *Schizochytrium* sp., have been demonstrated to have excellent fiber digestibility [162]. *Isochrysis* sp. and *Nannochloropsis* sp. fiber digestibility in rainbow trout was determined to be 96% and 38%, respectively [11]. Even though *Isochrysis* sp. contained more fiber than *Nannochloropsis* sp., the latter had a higher fiber digestibility, suggesting that the type of fiber (soluble or insoluble) is important for digestion. Compared to other nutrients, starch is a readily digestible food for fish and crustaceans [163].

The antiviral and antibacterial properties of β-glucan have been shown in humans, and it has also been shown in fish to have high antibacterial and immune-stimulating properties [164]. Furthermore, the types of carbon sources used and the metabolic mechanism employed are other important variables that affect the sugar concentration in microalgae [115]. The use of light has been shown to influence both algal growth and biomass composition during algal culture since light is an important energy source for photosynthetic activity [78,153]. For algal cultivation, the light intensity is normally between 200 and 400 mol photons m^2^/s. Nutritional restriction may be used to alter the metabolic process of microalgae and cause glucose accumulation [44].

## 3. Nutrients digestibility

It is important to understand how digestible feed components are used to calculate their nutritional value. Microalgal products are assessed for their nutritional digestibility before they can be used in aquafeeds. Many different types of microalgal organisms, from prokaryotes to eukaryotes, contribute to the wide variety. There has also been evidence of intra-species diversity in metabolic profiles. The nutritional and energy digestibility of various microalgal species varies due to differences in chemical composition and physical structure. Fish digestion of microalgae is influenced by the composition and rigidity of the cell [161]. In the prokaryotic (cyanobacterial) microalgae, peptidoglycan layers are present in the cell wall, while in eukaryotic microalgae, cellulosic layers occur [86,170,171]. Fish prefer microalgae with peptidoglycan (murein) layered cell walls over cellulose layered microalgae [86]. The rigidity of the cell wall also affects digestion. Thick-walled microalgae are less digestible than species with thin or no cell walls. *Desmodesmus, Nannochloropsis, Haemotococcus* and *Chlorella*, microalgae are with thick cell walls, while *I. galbana, Porphyridium cruentum*, and *D. salina* are with thin cell walls [85,110,172].

However, certain proteins may still alter the digestion of microalgae. Unwanted trypsin inhibitor, an enzyme that inhibits proteolytic enzymes, may cause poor digestion of *Nannochloropsis* sp. Some marine microalgae include lipase inhibitors that may affect lipid digestion [87,173,174]. Microalgae are made up of non-starch polysaccharides and fibers, making them difficult to digest [11]. Polysaccharides that are not made of starch, such as cellulose, gums, pectins, and hemicelluloses, are usually difficult to break down [170,175,176]. Digestive enzymes are absent in certain fish species, such as Nile tilapia, which cannot break down the beta glycosidic bonds found in non-starch polysaccharides [177]. Undigested carbohydrates are transported quickly by the digestive tract, absorb proteins, and reduce protein digestibility [178,179]. Fiber concentrations were shown to have a negative relationship with organic matter, protein, and carbohydrate digestibility [147,161]. This hypothesis has not been supported by any other study. Therefore, more research is needed to link fiber content with nutrient digestion [161].

In rainbow trout, *Isochrysis* sp. absorption of nutrients was shown to be superior to *Nannochloropsis* sp [11]. Fiber and other anti-nutrients also reduce proteolytic and amylase activity and digestibility [180,181]. Other variables can impact the digestion of a microalgae diet. Exopolysaccharides can form stable complexes with proteins, inhibiting proteolysis [161]. Exopolysaccharides are exopolysaccharides that are released or remain attached to cells in an algal culture [161]. Phenolic chemicals found in plants and seaweed can precipitate proteins. Although microalgae have a modest phenolic content (0–20 mg GA/g-DW), plant phenolic substances in the diet could impact microalgal protein digestion [182–184]. The digestibility of amino acids may be impaired. Furthermore, differences in physiology between fish species cause different digestibilities for the same microalgae [86]. Fish species vary in their digestive enzyme profiles, in addition to their physiology. Only a few fish, such as the Rohu (*Labeo rohita*), have the enzymes required to break down cellulose [185].

### 3.1 Bioaccumulation of heavy metals

During cultivation, microalgae cells can absorb and accumulate substantial amounts of heavy metals (HMs) [78,186–188]. Several studies have examined the bioaccumulation of HMs in microalgal biomass due to the existence of negatively charged functional groups on the surface of microalgal cells [189–191]. HMs absorbed by microalgae include predominantly arsenic (As), cadmium (Cd), copper (Cu), chromium (Cr), lead (Pb), mercury (Hg), nickel (Ni), and lanthanum (La). In fact, both living and non-living microalgae are effective in absorbing HMs from the environment and water [21,191]. Non-living microalgae can accumulate Cd, Cr, Hg, and Pb at 59, 98, 36, and 131 mg/g, respectively. Non-living algal biomass has a strong capacity to adsorb HMs primarily due to their surface functional groups [192].

When microalgal biomass containing concentrated HMs is fed to fish, harmful compounds can migrate up the food chain. Certain strategies can be employed to avoid bioaccumulation of HMs in the food chain when microalgae are used as aquafeed. The amount of HMs in the culture media should be monitored regularly during microalgae culture. If HMs concentrations exceed the EPA<apos;>s guidelines, the culture medium must be treated or relieved. This method may avoid the bio-accumulation of HMs in the food chain. Before using microalgae as fish food, it is possible to eliminate HMs from them. HMs desorption may occur through pH-induced desorption or metal-chelating agent treatment. The pH of algal cells must be lowered to the isoelectric points of functional groups on their surfaces to neutralize their surface charge [193]. Heavy metals adsorbed on the surface of algae might be combined with metal chelating agents to facilitate their desorption. HMs have been successfully desorbed from microalgae biomass using these two approaches [193–196]. When HMs are attached to the surface of algal cells rather than within algal cells, desorption techniques may be used to remove them from the biomass of microalgae. In contrast to the accumulation of HMs inside algal cells, microalgal cells can be treated by desorption to remove HMs adhering to the surface. Therefore, after desorption, HMs content in algal cells should be evaluated to determine whether microalgae can serve as aquafeed.

### 3.2 Deficiency in digestibility

Since microalgae have a high starch concentration, replacing the biomass of microalgae with a fish meal reduces digestibility. Furthermore, aquatic animals must dissolve the cellulose-rich cell walls of algae before consuming their nutritional components. For these two challenges, meals supplemented with microalgae- in fish and shrimp culture are poorly absorbed [197]. Dietary wheat starch levels of 20% in juvenile largemouth bass (*Micropterus salmoides*) not only impeded weight gain but also produced oxidative stress and impaired innate immunity. Fish offered 5% and 10% starch diets showed significant weight gain, growth rate, protein efficiency ratio, and feed conversion ratio when compared to fish-fed 20% starch diets [198]. It is generally recognized that omnivore fish digest carbohydrates are better than carnivorous fish, although they exhibit significant anatomical and physiological differences in their digestive systems [198,199]. Another study found that omnivorous fish have various digestive capacities [20]. Rather than raw biomass, defatted microalgae biomass is often used as a supplement to fish feed in the fishing sector. After oil extraction, the starch content in the biomass could be increased. The microalgae biomass must be effectively managed to avoid an excessive starch content. The apparent digestibility coefficients (ADCs) of macronutrients, amino acids, and fatty acids were studied in freshwater (*Arthrospira, Chlorella*) and marine microalgal (*Schizochytrium*) components in Nile tilapia [85]. Compared to *chlorella, Arthrospira* exhibited significantly higher ADCs of crude protein and all EAAs (86%), which corresponded well with the reported values for fishmeal and plant feeds. *Schizochytrium* had the highest DHA content, as well as the highest ADCs for lipids (total PUFA 98%), ω-3 (98%), and ω-6 (92.4%), as well as the maximum digestibility of DHA. *Spirulina* and *Schizochytrium* were shown to be effective protein substitutes for tilapia diets, while *Schizochytrium* was discovered to be a good LC-PUFA supplement [200].

For microalgal cells to be preserved, they usually need to be spray-dried after they have been removed from the growing reactor. Furthermore, microalgal biomass is dried in a variety of ways, such as sun drying, drum drying, and oven drying. However, the microalgae cell wall remains intact in these circumstances, indicating that digestion is restricted [87,201,202]. A cellulose-rich cell wall causes poor digestion of microalgae-supplemented fish diets [203]. The cell wall protects the algal cell and its intracellular components during growth. It is necessary to disrupt the cellulose structure of the cell wall in order to access the intracellular contents. Due to a lack of intestinal flora, several species of fish are unable to digest non-starch carbohydrates effectively [204,205]. Therefore, cellulose cannot be digested by fish, and the structure of the cell walls of microalgae cannot be effectively dissolved by many species of fish. As a consequence, aquatic organisms will not be able to consume nutrients found in algal cells. To improve the digestibility of microalgal biomass for use in fish/shrimp aquaculture, certain pretreatment techniques could be applied. Microalgae, for starters, contain starch, which may be separated before using algal biomass as aquafeed. Second, destroying the cell wall may cause the nutritional components of the microalgae to be released [206]. Enzymatic digestion and physical treatment have been shown to destroy microalgae cell walls effectively [206,207]. Microalgae cell walls are made up of pectin, hemicellulose, cellulose, and glycoproteins. In algae, enzymes such as glucosidase, cellulase, hemicellulase, xylanase, and exoglucanase break down the cell wall [206].

To improve nutrient digestibility, biomass can be pretreated/processed using pasteurization, freeze-drying, bead milling, high-pressure homogenization, pulse electric field, microwave, chemical, and enzymatic treatments [208]. During biomass processing or pretreatment, the rigid cell wall of algae is broken down, releasing internal nutrients that can be digested and absorbed by fish. According to studies, the processing of specific microalgae is linked to improved digestibility in certain fish [87,209,210]. Physical treatment processes such as sonication, the beating of beads, and freezing can be used to damage the microalgal cell wall. The cost of the aforementioned methodologies should be used to determine their viability in a real-world application [4]. The grinding of *Tetraselmis* sp. beads, for example, increased protein digestibility in European seabass by 20% compared to untreated cells [87]. Pretreatment with bead milled microalgae rather than whole biomass increased the digestibility of amino acids such as phenylalanine and aspartic acid in European seabass, but not the digestibility of essential amino acids [87]. *Nannochloropsis* and *Chlorella* cell walls were broken by bead milling for 10 minutes, which could release nutrients and improve digestibility [86,209,211]. For European seabass, enzyme processing improved the protein digestion of *Nannochloropsis* sp. and *Chlorella* sp., as well as energy digestion of *Nannochloropsis* sp. *Tetraselmis* sp., and *Chlorella* sp., by 14%, 11%, and 40%, respectively [87]. Due to the fact that the degree of cell rupture differs between species, nutritional accessibility can vary despite equal processing conditions [212].

### 3.3 Anti-nutritional factors

Anti-nutritional factors (ANFs) are biological components that affect gastrointestinal and metabolic function in animals and humans. These ANFs were detected in vegetable soybean and peanut proteins together with phytic, lectin, and tannic acids. Due to the increased nature of ANFs, protein meals or peanut meals cannot be used in aquafeed [213]. Microalgae have been shown to contain a variety of ANFs, such as *Tetradesmus obliquus, Kirchneriella lunaris*, and *Pseudokirchneriella subcapitata* [214,215]. Previous studies indicate that the tannic acid levels of *S. maxima* (6.86 mg/g) and *C. vulgaris* (1.44 mg/g) were comparable [216]. High absorption of microalgae may negatively affect fish growth because of antinutritional components in microalgae. ANF-supplemented aquafeed reduced liver enzyme activities and reduced intestinal brush border enzymes in juvenile Japanese seabass (*Lateolabrax japonicus*) [217].

It would be beneficial to discover and study anti-nutritional components in microalgae to increase their incorporation into fish diets. A pretreatment strategy for microalgae should also include the scavenging of anti-nutritional factors. Many studies have demonstrated effective ways to eliminate the anti-nutritional components from soybean meals [218,219]. Antinutritional chemicals may be removed by specific microorganisms through their activities. When *Aspergillus sojae* and/or *Aspergillus ficuum* are used in conjunction with solid-state fermentation, the phytic acid was reduced by 53.27% to 73.16%. Enzymes produced from microorganisms may also be used to remove certain anti-nutritional substances [220]. Another study found a maximum tannin removal rate of 73%. If antinutritional components are identified in microalgae, different physical treatment methods, including extraction, frying, blanching, and soaking, may be utilized to minimize their concentration. The pretreatment treatments described above have been shown to significantly decrease ANFs in a variety of vegetable proteins [221–224]. For example, extrusion cooking with optimal barrel temperature, extruder speed, and moisture content removed 61.25 % of tannin in linseed meals [224]. Eventually, these technologies may be utilized to pre-retreat microalgae to eliminate anti-nutritional components and surge algal biomass assimilation into fish diets, which would be beneficial in the long run.

### 3.4 Challenges in microalgae biomass harvesting and processing

Most aquafeed substitutes microalgae biomass for fish meal. Microalgae cultivated in a culture medium should be harvested and dried, and the moisture adjusted before being used to make algae biomass-supplemented fish feed pellets. Centrifugation, sedimentation, and filtration are all processes used to collect suspended algae cells in a culture medium. The collection of algae biomass accounts for 30% of microalgae production costs. In addition to that, flocculation harvesting incorporates aluminum into biomass, making microalgae-based aquafeed based on microalgae undesirable [225]. Drying wet microalgae (with a moisture content of 70–90 %) is required before the algae can be delivered to the feed factory. This is an energy-intensive, time-consuming, and costly technique [226]. Managing the moisture content of dehydrated microalgae biomass is important throughout the pelletization process.

Certain alternatives have been proposed and thoroughly explored to overcome the aforementioned concerns. The development of more cost-effective biomass harvesting methods is an essential first step. Co-cultivation of a filamentous fungus with microalgae previously resulted in fungal-algal pellets [15,227]. As a consequence, biomass collection has become a passive process that does not require human interaction. Several fungal strains, including *Mucor circinelloides* and *Aspergillus oryzae*, produce high-value components when cultured with microalgae, which can improve the nutritional content of biomass [228,229]. Microalgae immobilization techniques have also been implemented to minimize collection costs. After growing in the substratum, a biofilm<apos;>s biomass may be scraped off using scrapers. Immobilized microalgae are more economical to harvest than suspended microalgae [230]. Microalgae can be used to make an eco-friendly closed or semi-closed food chain aquaculture system. Zooplankton is the main food source for fish and shrimp in this environment. In addition to breaking down animal waste, microalgae can also transform it into valuable components [15,231]. Therefore, harvesting and drying can be avoided, and microalgae can be used to improve aquaculture water quality. Therefore, this unique approach to microalgae aquaculture is more economically feasible while still addressing the objectives of a circular economy.

## 4. Economic and environmental feasibility

To develop algae-based products as a possible source of food or feed for humans and animals, their economic feasibility and long-term viability must be improved. The Techno-economic assessment (TEA) and life cycle assessment (LCA) methods can analyze both the manufacturing route and the technical process of R&D activities to achieve commercial and environmental viability [232]. Based on six potential alternative sites, a techno-economic study was conducted on the entire life cycle of a 100-hectare microalgal production plant. Due to improved photosynthetic efficiency and photobioreactors with shorter light paths, it has been determined that the cost of algal production in Spain is approximated at 3.4 euros per kilogram of dry biomass with a predicted decrease to 0.5 euros per kilogram in ten years. The production of high-value metabolites (such as pigments) could generate 657 million euros in profits over the next 15 years (or more) [233].

Biotechnology can be used to create bio-based products, bioenergy, food, and feed while improving ethical and environmental sustainability, optimizing production processes, and lowering costs. Furthermore, to generate biomass, certain microalgal species have been shown to thrive in complex organic waste streams (digestate, wastewater, etc.) and to remove nutrient pollutants (P, N, and other toxins) [234–237]. As a consequence, integrated biorefineries based on microalgal biotechnology could recover some agricultural by-products while reducing disposal costs. Compared to monoculture cultivation, heterotrophic cultivation is cost-effective since it takes less land and investment, consumes more energy and carbon, and has a lower cost of downstream processing [237,238]. Microalgae protein has a significant environmental impact because the drying process is so energy-intensive. In contrast to beef and pork, the LCA in autotrophic and heterotrophic algae cultures resulted in more ecologically sustainable products derived from heterotrophic cultivation [239]. When using hydrolyzed food waste to obtain carbon in microalgal production, the environmental benefit could be 4.5 times greater, making it much more eco-friendly among protein sources.

## 5. Current concerns with microalgae in aquafeed

The high cost of microalgae production continues to be a barrier to aquaculture. Microalgae have the potential to be used economically by cutting the costs of production and distribution [85,240]. Microalgae are difficult to dry and pelletize, and incorrect drying can alter their nutritional and physical qualities, which in turn reduces their use as feed. Some microalgae (such as *Chlorella*) have thick cell walls that hinder nutrient uptake. Some microalgae (e.g., *D. tertiolecta*) have extracellular polysaccharides that might interfere with nutritional absorption. Poor digestibility and significant salt buildup in marine microalgae species used as fish feed might cause problems. Algae can only provide 10–15% of dietary protein requirements in test diets without affecting development or food consumption. Several microalgae have a high carbohydrate and low protein content because their tough cell walls inhibit the digestion of fats and proteins [163].

Due to the high concentrations of trace elements and toxins found in microalgae biomass, it is not recommended for use in aquafeeds. Protein left behind after fat is extracted for the generation of biofuels is often proposed for use in animal feed [241]. It is possible that microalgae used to produce biofuels are not suitable for feeding and that the demand for low-cost fuel production could result in toxic protein residues [242,243]. It is preferable to first utilize algal biomass for higher-value products, such as aquafeed, and then use any remaining chemicals for biofuel production. This means a high-value product-first philosophy [244,245]. The lack of substantial amounts of microalgal biomass may hinder the growth of the aquafeed sector. Massive amounts of microalgal biomass for the aquafeed industry require successful large-scale algal growth of commercially relevant microalgae species [246].

## 6. Perspective and future direction

The cell walls of microalgae vary in content and structure. This emphasizes the need to screen for commercial strains that can be readily handled for cell disruption. Only a few microalgal species have been digestible tested. Several microalgae and cyanobacteria species, including *Anabaena* sp. and *Nostoc* sp., have not been tested for digestibility by fish or other aquatic species. *D. salina*, for example, does not have a cell wall, which can improve digestion of cellular metabolites. It is also critical to explore the impact of environmental variables and stress on the chemical composition of microalgae. For example, nitrogen deficiency could cause a buildup of PUFA and starch, which fish can consume. The metabolic response of microalgae to stress, such as nitrogen deficiency, differs between species. In certain strains, nitrogen deficiency lowers protein levels while increasing carbohydrate levels [247]. Optimal stress management and species selection are required to achieve a desirable biochemical profile and digestibility.

In addition to the availability of microalgal biomass at a reasonable price, microalgal and aquafeed producers must address substantial variability in the proximate composition, digestibility, and growth conditions. Aquaculture requires a diverse range of microalgal species. Microalgal species with better nutritional or growth properties may be more efficient. The nutritional value of various microalgae should be investigated. This means that the cell wall must be broken down to make the algal elements available to digestive enzymes [147,211,248]. However, further processing stages may increase costs. To improve fish health, microalgal material must be tested for harmful chemicals prior to commercialization. In addition to nutritional content and digestibility, the processing requirements of industrial production lines for compound feed must also be considered. The composition of extruded fish feed is acknowledged to be one of the most critical elements that impact physical quality [249]. Microalgae were examined in a recent study to physically determine their effects on extruded fish feed, but further research is necessary [208].

Due to the explosive growth of the aquaculture industry, it is important to study the digestibility of microalgae. So far, digestibility has been examined only for salmonids, tilapia, seabass, and African catfish. During feed screening trials, microalgae should be evaluated for anti-nutritional factors, such as digestion enzyme inhibitors (e.g., caulerpenyene, a terpene) and nutrient bioavailability factors. Studies on macronutrients, such as lipids, carbohydrates, protein, and energy, have previously focused on microalgae. However, specific classes of these macromolecules have been shown to impact digestion. Therefore, it is essential to determine the digestibility and composition of microalgae for certain species of fish.

The energy-intensive harvesting stage of microalgae biomass results in high production costs. Microalgal biorefinery approaches might reduce microalgal feed component manufacturing costs. This approach extracts high-value biochemical components from biomass, such as lipids and carotenoids, which are then used in fish and animal feed. Supercritical fluid extraction and organic solvent extraction in combination are effective for defatting biomass. Although defatted biomass from the aforementioned biorefinery processes has been studied, no reports on saponified biomass have been published. The saponification-based biorefinery produces carotenoids and other value-added microalgae products. The residual biomass may be used as fish feed. Therefore, the residual biomass of biorefinery methods must be examined for digestion in fish.

## 7. Conclusions

Due to the depletion and pollution of marine resources, aquaculture is increasingly turning to microalgae as a substitute for fishmeal. Astaxanthin, polyunsaturated fatty acids (PUFAs), and phycocyanin are just a few of the high-quality components found in microalgae. There is little doubt that these high-quality constituents can improve an animal<apos;>s immunological response, increase its survival rate, and help it gain weight. The biosynthesis of astaxanthin, PUFAs, and phycocyanin in microalgal cells is technically feasible to produce high-quality biochemical products. Thanks to significant bioengineering technology advances, microalgal biomass and bioproducts such as aquafeed are no longer just a theory. In addition, the culture medium should be regulated to avoid the accumulation of HMs in the biomass. To make microalgae suitable for aquafeeds, the cell wall of the algae must be broken down to release intracellular nutrients and scavenge nutrient-hostile chemicals. A co-culture of microalgae with filamentous fungi is being developed to facilitate harvesting and minimize microalgal biomass costs. Eco-friendly aquaculture systems based on microalgae, zooplankton, and fish/shrimp are also gaining importance in the circular bio-economy. A critical analysis revealed that bioengineering tools and strategies could significantly improve the development of aquaculture-based food production. Additionally, the study also provides new insights into the challenges and potential breakthroughs regarding microalgae biomass production and bioproducts as sustainable ingredients in the future.
